# In silico evidence of de novo interactions between ribosomal and Epstein - Barr virus proteins

**DOI:** 10.1186/s12860-019-0219-y

**Published:** 2019-08-15

**Authors:** Edmund Ui-Hang Sim, Shruti Prashant Talwar

**Affiliations:** Faculty of Resource Science and Technology, Universiti Malaysia Sarawak, 94300 Kota Samarahan, Sarawak Malaysia

**Keywords:** Epstein-Barr virus, Ribosomal proteins, EBNA1, Protein-protein interactions, Computational prediction

## Abstract

**Background:**

Association of Epstein-Barr virus (EBV) encoded latent gene products with host ribosomal proteins (RPs) has not been fully explored, despite their involvement in the aetiology of several human cancers. To gain an insight into their plausible interactions, we employed a computational approach that encompasses structural alignment, gene ontology analysis, pathway analysis, and molecular docking.

**Results:**

In this study, the alignment analysis based on structural similarity allows the prediction of 48 potential interactions between 27 human RPs and the EBV proteins EBNA1, LMP1, LMP2A, and LMP2B. Gene ontology analysis of the putative protein-protein interactions (PPIs) reveals their probable involvement in RNA binding, ribosome biogenesis, metabolic and biosynthetic processes, and gene regulation. Pathway analysis shows their possible participation in viral infection strategies (viral translation), as well as oncogenesis (Wnt and EGFR signalling pathways). Finally, our molecular docking assay predicts the functional interactions of EBNA1 with four RPs individually: EBNA1-eS10, EBNA1-eS25, EBNA1-uL10 and EBNA1-uL11.

**Conclusion:**

These interactions have never been revealed previously via either experimental or in silico approach. We envisage that the calculated interactions between the ribosomal and EBV proteins herein would provide a hypothetical model for future experimental studies on the functional relationship between ribosomal proteins and EBV infection.

**Electronic supplementary material:**

The online version of this article (10.1186/s12860-019-0219-y) contains supplementary material, which is available to authorized users.

## Background

Epstein-Barr virus (EBV), a type of herpesvirus that is common in human, has been known to be associated with cancers such as Hodgkin’s lymphoma, Burkitt’s lymphoma, gastric cancer, and nasopharyngeal carcinoma [1]. At the same time, the roles of ribosomal protein (RP) genes in tumourigenesis of various cancers, mainly via their extraribosomal functions, have been widely revealed [2, 3]. Despite this, there is limited understanding of the interactions between EBV and human ribosomal proteins in condition of carcinogenesis, although such interactions do exist. The EBV Nuclear Antigen 1 (EBNA1) protein has been found to bind Ribosome Protein L4 (uL4) in a complex that includes Nucleolin (NCL), and has the functional relevance of an EBV-mediated tumourigenesis [4]. Ribosomal protein s27a (eS31), on the other hand, interacts with and regulates the stability of EBV-encoded latent membrane protein 1 (LMP1) by inhibiting proteasome-mediated ubiquitination [5]. These findings represent a scant insight of the complete repertoire of functional interactions between the proteins of EBV and ribosome, of which is yet to be fully explored. Protein-protein binding assays and associated functional studies of the 80 known human RPs and 9 EBV proteins will undoubtedly be a resource-intensive and time-consuming endeavour if experimental approach is the only means of study.

As such, computational approaches for predicting host-virus protein interactions can provide viable hypothetical model for identifying potential protein-protein interaction scenarios to benefit future experimental design on the study of EBV-RP interactions. A valid in silico method for this purpose is the structural similarity-based strategy from the sequence-to-structure-to-function paradigm [6]. This approach is based on using protein structure information for prediction of interactions, and the assumption that proteins with similar structures will tend to share interaction partners [6, 7]. It has been employed previously for the prediction of several virus-human host interactions [8–10] and also for plausible protein partners of some RPs [11, 12]. Hence, this strategy forms the basis of our study here to computationally predict interactions between proteins of EBV and ribosome. By this, a multitude of potential interactions were predicted among 27 human RPs and four EBV proteins (EBNA1, LMP1, LMP2A, and LMP2B). Various functional significance and associated pathways underlying these interactions have been suggested. Molecular docking analysis on selected EBV protein and RPs reveal simulated interactions between EBNA1 protein with each of the four RPs of eS10, eS25, uL10 and uL11. These de novo interactions derived from in silico evidence will be vital insights for deciphering the mechanisms of EBV-associated oncogenesis where human/host RPs played a cooperative role.

## Results

### In silico identification of RPs that interact with EBV proteins

**Fig. 1:**
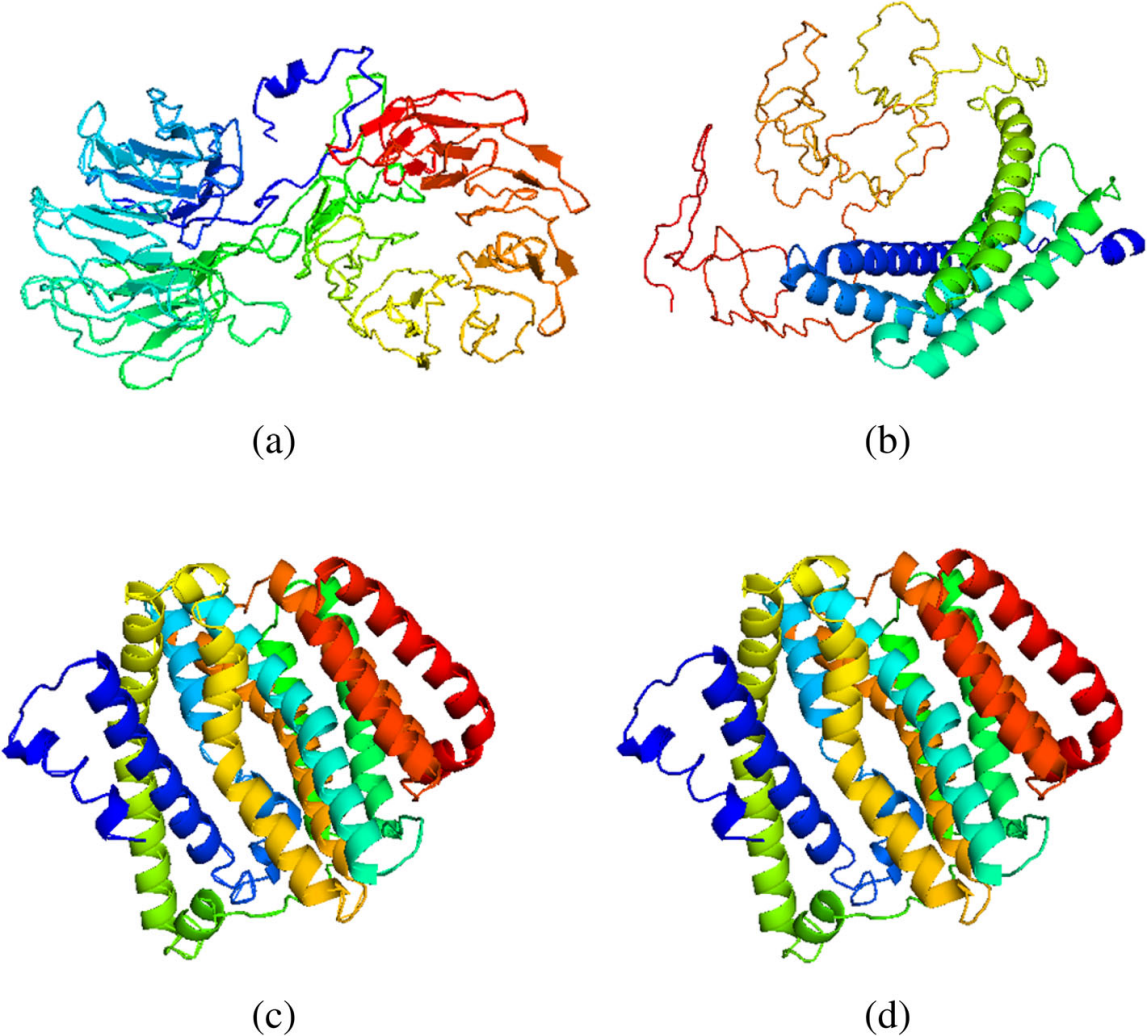
Ribbon representation of the 3D models predicted for the EBV proteins, **a** EBNA1; **b** LMP1; **c** LMP2A; and **d** LMP2B, after structure refinement. Model images were generated in PyMOL molecular graphics system (version 1.8). Each is coloured from N-(blue) to C-terminus (red) as a spectrum

The best 3-D structural models of EBV proteins generated using I-TASSER (Fig. 1) were selected based on the qualities of geometrical and stereochemical parameters (Table 1). Subsequent structure matching procedure by DaliLite revealed 53, 138, 27, 87 and 62 human proteins (hEBV) with similarity to EBNA1, LMP1, LMP2A, LMP2B and BARF1, respectively.

**Table 1:** Quality estimations of the EBV protein models, after structure refinement

Model Quality Evaluations	EBV Protein Models			
	EBNA1	LMP1	LMP2A	LMP2B
RAMPAGE				
Residues in favoured region (%)	87.80	86.50	94.70	92.80
Residues in allowed region (%)	9.70	12.20	3.50	5.90
Residues in outlier region (%)	2.50	1.30	1.90	1.30
ERRAT	86.34	67.46	94.23	93.43
Verify 3D	87.52	83.16	70.90	66.74
QMean	0.187	0.324	0.234	0.24

Further analysis using data from HPRD and IntAct, demonstrated these hEBV to be associated with nearly five thousand human proteins. From these, we narrow down the list to those that are RPs (Table 2). These 34 RPs are considered as potential interacting partners of the EBV proteins.

**Table 2:** Predicted interactions between EBV proteins and ribosomal proteins. Names of RPs are based on new nomenclature system [13]

EBV Proteins	Inferred Ribosomal Protein Interactors
EBNA1	eS10, uS9, eS25, uL11, uL10, and P2.
LMP1	uS3, eS1, eS4, eS6, uS17, uS15, uS19, eS25, eS27, eS28, uL4, uL18, eL6, uL30, uL2, uL1, eL13, eL19, eL28, eL30, eL31, eL39, uL10, P2, RPS4X and, RPL39L.
LMP2A	uS5, uS3, eS7, uS9, uL18, eL6, uL1, eL19, eL31, uL10, and P2.
LMP2B	uS3, eS1, eS4, eS6, uS17, uS15, uS10, eS31, uL4, uL18, eL6, uL30, uL2, eL30, eL33, uL10 and, RPS4X.
BARF1	eL14, and eL41.

### Assessment of the predicted interactions

An extensive search through the IntAct database led to the retrieval of 143 experimentally determined PPIs between EBV and Human proteins. Of these, 14 were also present in the current study as shown in Table 3. Given that even large-scale experimental protein interaction studies typically show little overlap in their results [8], it was promising to note that nearly 10% of known interactions were also present in the predicted PPI.

**Table 3:** Experimentally determined EBV – host protein-protein interactions which were also predicted in the present study

EBV protein	Human protein	Experiment type	Reference
EBNA1	SRPK2	two-hybrid array	[14]
	CSNK2A1	anti-tag co-immunoprecipitation	[15]
	CSNK2B	tandem affinity purification	[14]
LMP1	RABAC1	two-hybrid	[16]
	UBE2I	anti-bait co-immunoprecipitation	[17]
	TRADD	anti-bait co-immunoprecipitation	[18]
	UBQLN1	two-hybrid array	[14]
LMP2	PDGFRB	tandem affinity purification	[14]
	CD44	tandem affinity purification	[14]
	PSMA3	tandem affinity purification	[14]
	EMD	tandem affinity purification	[14]
	ATP2C1	tandem affinity purification	[14]
	PSME1	tandem affinity purification	[14]
	PSME2	tandem affinity purification	[14]

### Prediction of biological processes and molecular functions of targeted RPs

**Fig. 2:**
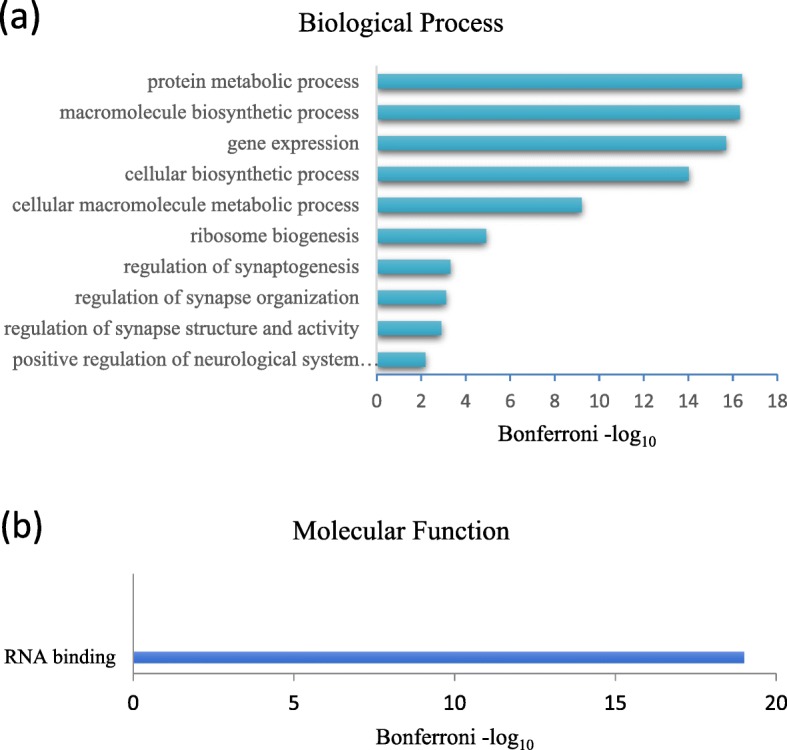
Gene Ontology (GO) term enrichment of ribosomal proteins interacting with EBV proteins. The enriched **a** GO biological process terms, and **b** GO molecular function terms have been depicted. Bonferroni corrected *p*-values were transformed by log_10_

Annotations based on both Molecular Function (MF) and Biological Process (BP) categories, derived from GO term enrichment analysis via DAVID, provided limited functional scenarios of the predicted RPs-EBV proteins interaction (Fig. 2). The GO term ‘RNA binding’ was the only significantly enriched MF category term that was retrieved. Overall results reflect the notion that ribosomal proteins here are likely associated with processes relevant to EBV infection and/or oncogenesis.

### Pathway data of targeted RPs

The statistically significant enriched pathways in the predicted PPIs between EBV proteins and targeted RPs is summarised in Table 4. Our prediction analysis showed enrichment for pathways associated with viral infection strategies (such as viral translation) and oncogenesis (such as Wnt signaling, EGFR signaling).

**Table 4:** The top ten significantly enriched pathways of targeted ribosomal proteins in the predicted PPI, based on DAVID and KOBAS analysis

Platform	Pathway database	Pathway names	*p*-value
DAVID	KEGG	Ribosome	2.68e-43
	Reactome	3′ -UTR-mediated translational regulation	2.07e-41
	Reactome	Influenza Infection	8.22e-37
	Reactome	Metabolism of proteins	6.79e-33
	Reactome	Gene Expression	3.96e-27
	Reactome	APC-Cdc20 mediated degradation of Nek2A	0.002563
	Reactome	Regulation of activated PAK-2p34 by proteasome mediated degradation	0.014244
	Reactome	Signaling by EGFR	0.014717
	Reactome	APC/C: Cdh1-mediated degradation of Skp2	0.026468
	Reactome	Signaling by Wnt	0.026468
KOBAS	Reactome	Viral mRNA Translation	4.96e-55
	Reactome	Peptide chain elongation	4.96e-55
	Reactome	Selenocysteine synthesis	5.58e-55
	Reactome	Eukaryotic Translation Elongation	6.99e-55
	Reactome	Eukaryotic Translation Termination	7.83e-55
	Reactome	Nonsense Mediated Decay (NMD) independent of the Exon Junction Complex (EJC)	1.26e-54
	Reactome	Formation of a pool of free 40S subunits	2.05e-54
	Reactome	L13a-mediated translational silencing of Ceruloplasmin expression	1.50e-53
	Reactome	GTP hydrolysis and joining of the 60S ribosomal subunit	1.61e-53
	Reactome	Nonsense-Mediated Decay (NMD)	3.61e-53

### Refined protein-protein interaction network

**Fig. 3:**
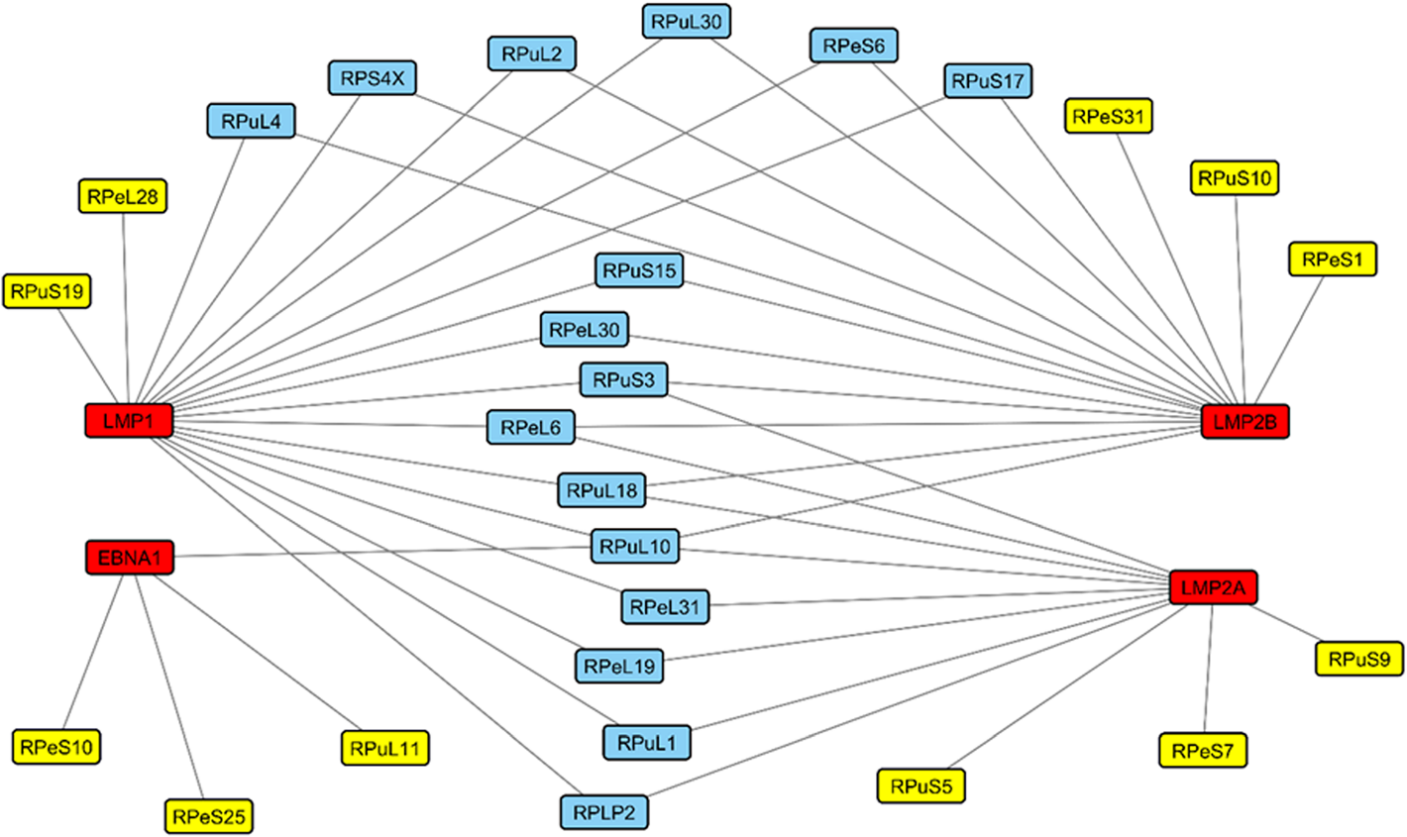
Predicted interactions between EBV proteins and ribosomal proteins. The red rectangles represent the viral proteins; (BARF1 protein had no interactors after CC filtering, and hence is not depicted). Yellow and blue rectangles represent host ribosomal proteins interacting with one or more than one viral protein, respectively. Network diagram was created using Cytoscape

Based on the theoretical assumption that direct interaction between proteins requires their presence in the same cellular compartment, our interaction dataset was analysed to discern protein subcellular co-localization. Protein pairs which did not contain shared GO cellular component (CC) terms were considered false positive and excluded. Our result of a refined PPI between EBV and ribosomal proteins is illustrated in Fig. 3. There are 48 predicted interactions between EBV proteins and the 27 human ribosomal proteins, after CC filtering. This eventual interaction network represents high-confidence predictions with coherent functional and biological attributes.

### Molecular docking

Docking assays were conducted for EBNA1 and its inferred ribosomal protein interactors eS10, eS25, uL10 and uL11. Prior to this, for the construction of 3D models for the four RPs, template proteins were selected by subjecting the amino acid sequences of the ribosomal proteins to NCBI PSI-BLAST [19] against PDB proteins, wherein two iterations were performed with default parameters. Amongst the hits, only templates with structures of expected value (E-value) better than threshold, maximal sequence identity and high structure resolution were chosen. Three strategies were employed to predict tertiary structure models.

**Fig. 4:**
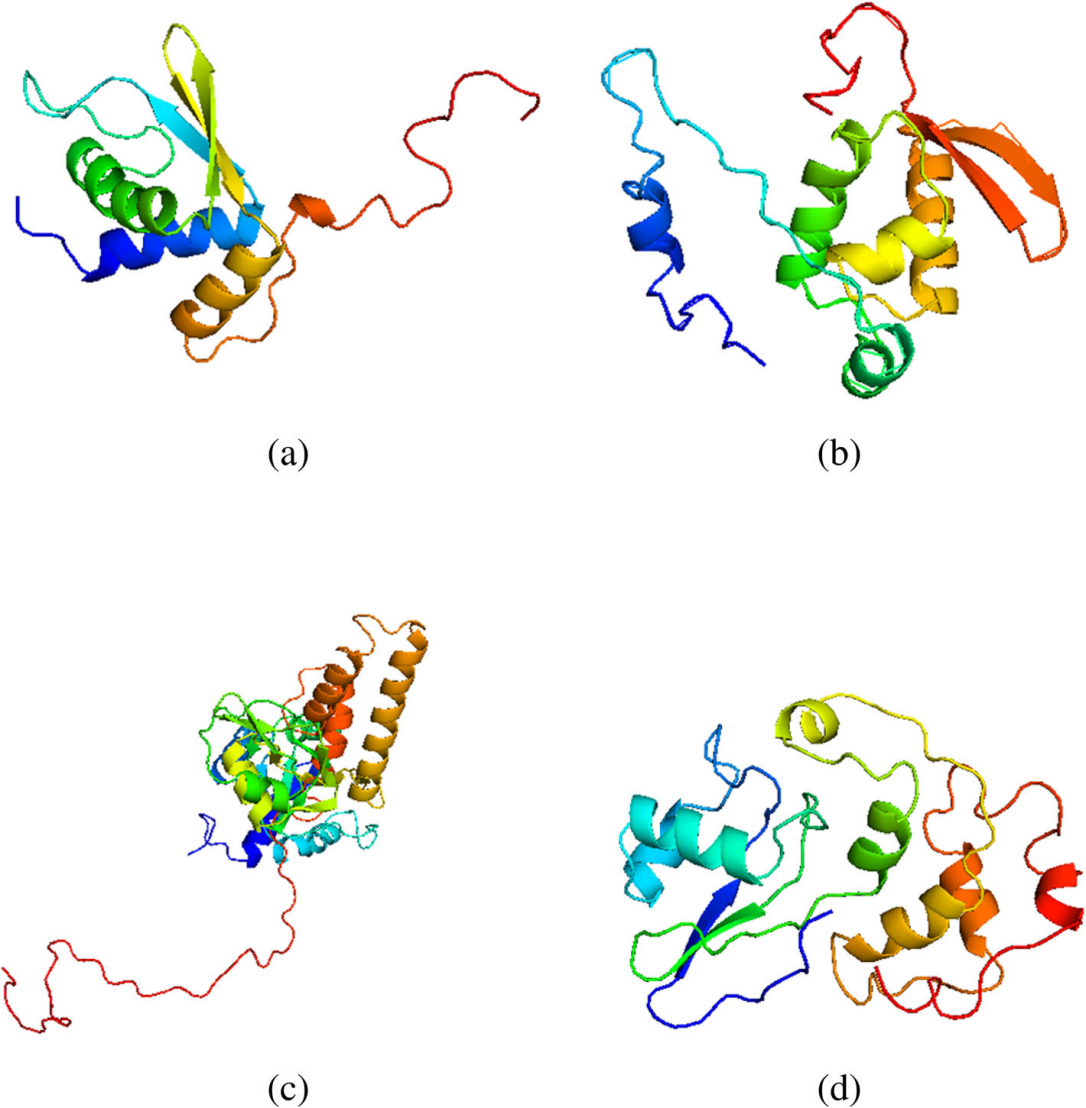
Ribbon representation of the 3D models predicted for each ribosomal protein, after structure refinement: **a** eS10, **b** eS25, **c** uL10, and **d** uL11. Homology models for eS10 and eS25 were generated by I-TASSER, for uL10 by RaptorX, and for uL11 by SWISS MODEL. Model images were rendered in PyMOL molecular graphics system (version 1.8). Each is colour-coded from N-(blue) to C-terminus (red) as a spectrum

In the SWISS-MODEL analysis, for the eS10 model, the Chain K of the eukaryotic ribosome structure (PDB ID: 3U5C) at 3.0 Å resolution with 54% identity and E-value 2e-54 was chosen. For the eS25 model, Chain 8 of the crystal structure of eukaryotic 40S ribosomal subunit in complex with initiation factor-1 (PDB ID: 2XZM) at 3.93 Å resolution with 32% identity and E-value of 5e-15 was selected. The uL10 model was predicted by choosing the structural template Chain M of the yeast 80S ribosome (PDB ID: 3O5H) at 4.0 Å resolution with 54% identity and E-value of 1e-124. Lastly, the uL11 model was generated using the template structure of ribosomal protein L11 from *Methanococcus jannaschii* (PDB ID: 5COL) at 2.25 Å resolution with 32% identity and E-value of 7e-55. Prediction analysis was also done using RaptorX and I-TASSER. After structure refinement, the best model was selected on the basis of quality assessment for geometrical and stereochemical parameters (Table 5). Consequently, the I-TASSER models were selected for eS10 and eS25, RaptorX model for uL10, and SWISS-MODEL model for uL11 (Fig. 4).

**Table 5:** Model quality estimations of the selected ribosomal protein models, after structure refinement

Model Quality Evaluations	Ribosomal Protein Models			
	eS10	eS25	uL10	uL11
RAMPAGE				
Residues in favored region (%)	90.20	91.10	94.90	94.80
Residues in allowed region (%)	8.60	7.30	4.80	3.90
Residues in outlier region (%)	1.20	1.60	0.30	1.30
ERRAT	92.70	100.00	98.17	98.59
Verify 3D	100.00	76.00	54.89	84.52
Q Mean	0.594	0.585	0.518	0.650

### EBNA1-eS10 docked complex

**Fig. 5:**
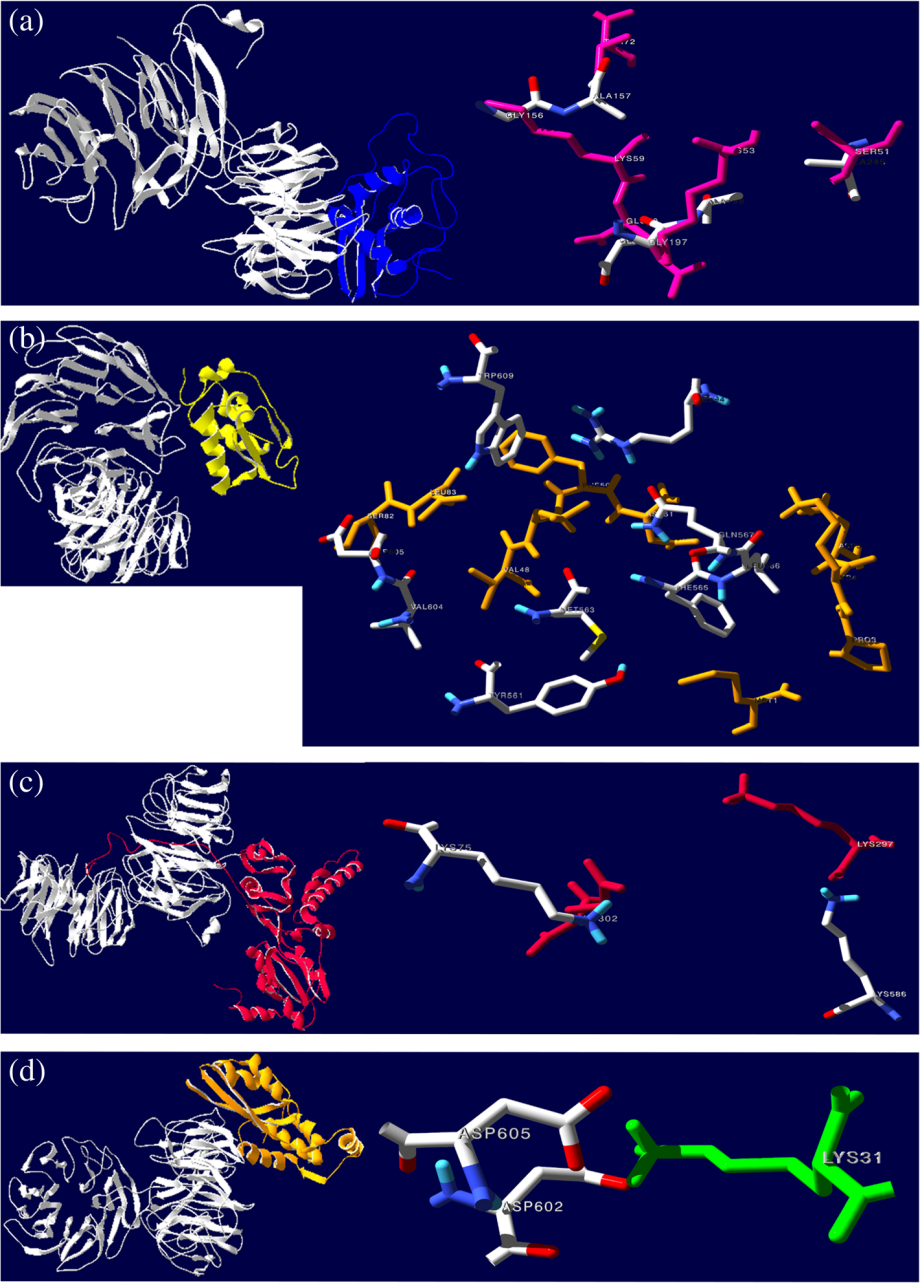
Docked models of, **a** EBNA1-eS10, **b** EBNA1-eS25, **c** EBNA1-uL10, and **d** EBNA1-uL11 complexes. Enlarged view of the contact residues area for each docked complex is shown on the right panel, wherein EBNA1 residues are illustrated in CPK colouring and the RP residues correspond to their respective colour on the left panel

Out of the docked conformations generated by ClusPro, potential EBNA1-eS10 complex was selected from the balanced category on the basis of higher cluster size and lowest energy, which were consequentially found to be 93 and − 1160.5 kcal/mol, respectively. The top ten solutions predicted and refined through PatchDock/FireDock reveals desolvation energy of − 1.80 kcal/mol and global free energy of − 0.31 kcal/mol for the best docked conformation. Thus, the energy profiles obtained from both servers indicated high interaction probability for EBNA1 and eS10. Interface of the individual EBNA1-eS10 complex were further analysed to identify residues in the interactions, which were within 3.5 Å of each other (Additional file 1: Table S1). Based on the maximum number of contact residues and visual inspection of interfacial region, the PatchDock model is selected as the most likely conformation to portray the EBNA1-eS10 complex (Fig. 5a). PIC examination of the binding site interface of EBNA1 and eS10 reveals interacting residues mainly involved in hydrophobic interactions (Additional file 1: Table S2).

### EBNA1-eS25 docked complex

Potential ClusPro-generated complex of EBNA1-eS25 that was selected has a high cluster size and low energy of 212 and − 784.2 kcal/mol respectively. The best docked conformation (predicted and refined by PatchDock/FireDock) has a desolvation and global free energy of 0.54 kcal/mol and 8.55 kcal/mol respectively. Despite a fair energy profile, the ClusPro binding mode demonstrated high interaction probability for EBNA1 and eS25. Interface analysis of the individual EBNA1-eS25 complex was conducted to identify residues in the interactions, which were within 3.5 Å of each other (Additional file 2: Table S3). Based on the maximum number of contact residues, and visual inspection of interface region, the ClusPro model is chosen as the most likely conformation for the EBNA1-eS25 complex (Fig. 5b). PIC analysis predicts only hydrophobic interactions of the interface residues (Additional file 2: Table S4).

### EBNA1-uL10 docked complex

Selected EBNA1-uL10 complex has a higher cluster size (44) and lowest energy (− 1243.3 kcal/mol) amongst the ClusPro-generated complexes. The best docked conformation from the top ten solutions predicted and refined via PatchDock/FireDock has a desolvation and global free energy of − 2.55 and − 0.18 kcal/mol respectively. Thus, the energy profiles obtained from both servers indicated high probability for EBNA1-uL10 interaction. In addition, interface analysis identified interacting residues within 3.5 Å of each other (Additional file 3: Table S5). Based on the maximum number of contact residues, and visual inspection of interface region, the ClusPro model (Fig. 5c) is selected as the most likely conformation. PIC examination of the binding site interface of EBNA1 and uL10 reveals interacting residues involved in hydrophobic and ionic interactions (Additional file 3: Table S6).

### EBNA1-uL11 docked complex

The selected ClusPro-generated EBNA1-uL11 complex has a higher cluster size (238) and lowest energy (− 946.4 kcal/mol) amongst other complexes. At the same time, the best among the top ten conformations predicted using PatchDock/FireDock has a desolvation and global free energy of − 0.11 kcal/mol and − 1.55 kcal/mol respectively. These energy profiles indicate the likelihood of the interaction between EBNA1 and uL11. The ClusPro model is selected based on the maximum number of contact residues and visual examination of interface region (Additional file 4: Table S7). The docked model and potential contact residues at the interface of the EBNA1-uL11 complex are shown in Fig. 5d. PIC assessment reveals hydrophobic and ionic interactions in the binding site interface of EBNA1 and uL11 (Additional file 4: Table S8).

## Discussion

Overall, we reveal 48 possible interactions between 27 RPs and four EBV proteins. Our computational strategies have allowed us to imply the functional significance of viral infection and oncogenesis as a result of these interactions. It is worth mentioning herein that these interactions were predicted on the basis of associations between the targeted EBV proteins and ribosomal proteins as disassembled individual proteins rather than as ribosome complexes. In the scenario of infection, survival of the virus (EBV, in our case) means that the host cell must be induced to be translationally competent at all times. This situation requires the persistent synthesis of viral and cellular proteins to ensure the viability of the virus and host [20]. Studies on infection by HSV-1 (a herpesvirus related to EBV) have found that the continued synthesis of ribosomal proteins is critical for maintaining viral persistence and latency [21, 22]. Indeed, our data demonstrates that the RPs predicted to interact with the EBV proteins is associated with cellular and macromolecular biosynthetic processes. It is conceivable that the RPs identified in the present study may support viral mRNA translation, in part, by making sure that the synthesis of ribosomal proteins remained sustained during latent EBV infection. However, whether complexes of EBV proteins and RPs are necessary factors behind these phenomena remains to be further explored.

The roles of RPs in tumourigenesis are widely known and partly explained [2, 3]. However, this paper is the first to provide insights into their interaction(s) with EBV proteins in EBV-mediated oncogenesis. Our pathway enrichment analysis reveals two notable pathways, the Wnt and EGFR signalling mechanisms, which could underlie this situation. Incidentally, evidence of Wnt pathway modulation by EBV is not uncommon [23–26]. Likewise, EGFR signalling is known to be targeted by the EBV protein, LMP1, to mediate transformation via LMP1-induced endocytosis and nuclear translocation of EGFR [27]. Although the precise role(s) of EBV-RP interactions in both pathways remains to be experimentally delineated, our in silico findings may provide some valuable insights. It is with this premise in mind that we selectively targeted EBNA1 (the only viral protein expressed in all EBV-associated tumours [60]) and its predicted RP interactors (eS10, eS25, uL10 and uL11) for further investigation (docking simulation). Evidently, all four RPs show significant likelihood of PPI with EBNA1, and provided de novo computationally relevant complexes.

The physiological significance of our predicted EBNA1-eS10 and EBNA1-uL11 complexes may be difficult to speculate based on literature. Mutation of *RPeS10* is evident in the congenital disorder of Diamond-Blackfan anaemia [28], and causes a deregulated 40S/60S ribosomal subunit ratio leading to sub-optimal protein synthesis [29]. In the case of uL11, its up-regulation is found in human hepatocellular carcinoma [30]. For all these diseases, EBV is an irrelevant factor.

Conversely, the postulated EBNA1-eS25 complex is more functionally relevant. EBNA1 interacts with the cellular ubiquitin specific protease (USP7/HAUSP) to destabilise p53 by competitively inhibiting USP7-p53 interaction [31–33]. eS25, on the other hand, binds to MDM2 and inhibits its E3 ubiquitin ligase activity, leading to p53 activation [34]. Could the EBNA1-eS25 interaction be an alternate or additional route for EBNA1-mediated destabilisation of p53? Indeed, here we provide a new perspective on possible explanation of tumour suppression loss in the event of EBV-induced tumorigenesis. The physiological relevance of an EBNA1-uL10 interaction with respect to oncogenesis can also be suspected. Ribosomal protein P0 (uL10) is an apoptosis-associated protein identified in a Burkitt lymphoma cell line [35], while EBV infection is strongly correlated with this cancer [1, 36]. Here, the possibility of EBV in deregulating apoptosis during oncogenesis is a notion worth investigating.

## Conclusion

Based on structural similarity-based prediction protocol, we have provided in silico evidence of 48 de novo biologically relevant protein-protein interactions among 27 ribosomal proteins and four EBV proteins. We have further postulated that the resultant complexes derived from these interactions may be associated with the functions of viral infection and oncogenesis. From focus molecular docking analysis, we derived four statistically feasible docked complexes between the EBV protein EBNA1 and each of its predicted RP interactors (eS10, eS25, uL10 and uL11). Only two of these complexes (EBNA1-eS25 and EBNA1-uL10) are suspected to have functional significance in EBV-mediated oncogenesis.

## Methods

**Fig. 6:**
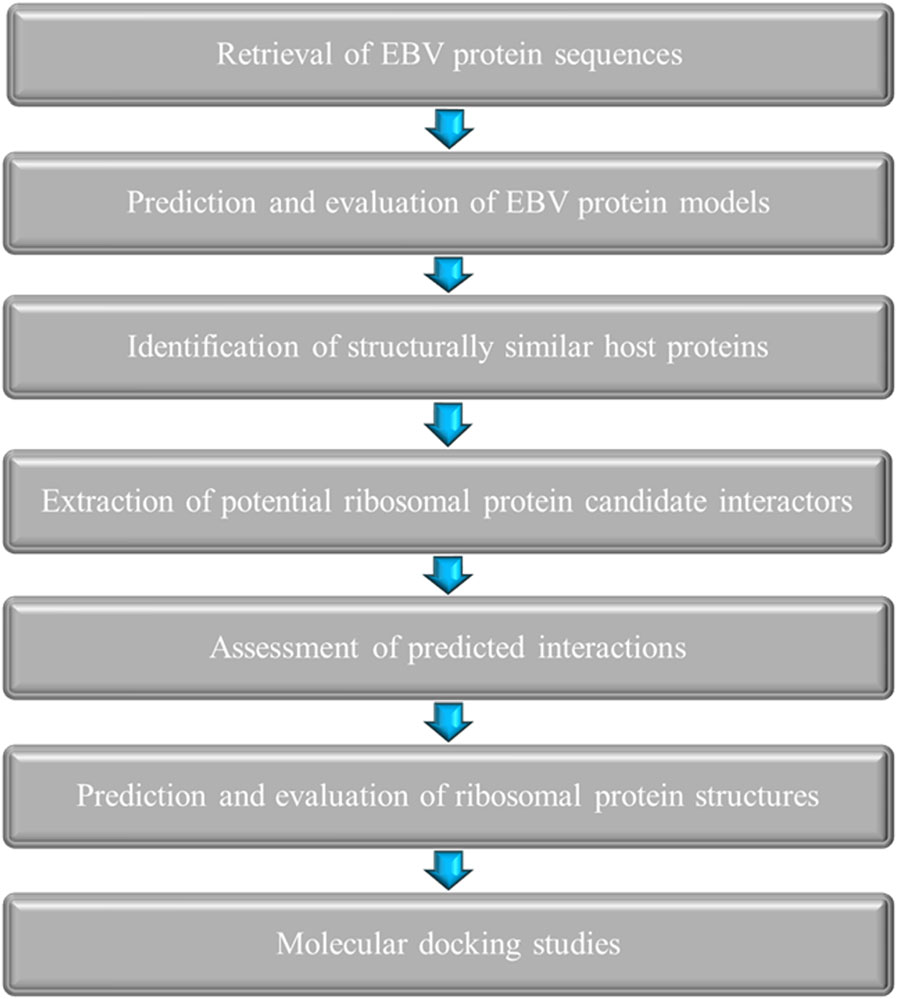
Schematic overview of the computational prediction of interactions between EBV proteins and human ribosomal proteins

The multi-step protocol which was implemented for the computational elucidation of interactions between EBV proteins and human ribosomal proteins has been delineated in a schematic overview in Fig. 6.

### Data sources

Experimentally verified full length crystal structures of EBNA1, LMP1, LMP2A and LMP2B are not available in Protein Data Bank (PDB), and hence were modeled using the I-TASSER platform [37, 38]. The protein sequences used for generation of tertiary structural models were acquired from the National Center for Biotechnology Information (NCBI) with accession numbers YP_401677.1 (EBNA1), YP_401722.1 (LMP1), YP_401631.1 (LMP2A), and YP_401632.1 (LMP2B). The available crystal structure of BARF1 (PDB ID: 2CH8) was retrieved from PDB. Each of the EBV protein (known or predicted) was compared with proteins of known structures for structural similarities using the DaliLite webserver v. 3 [39, 40]. The known protein-protein interactions among human proteins were assimilated from IntAct v. 4.1.8 [41] and Human Protein Reference Database (HPRD) Release 9 [42]. Tertiary structural models are visualized in PyMOL molecular graphics system v. 1.8 [43].

### Identification of structural similarity among EBV and host proteins

The computer-generated tertiary structures of EBV proteins were refined to reduce side chain steric clashes and bond length errors using the ModRefiner programme [44]. Model quality assessments were conducted by RAMPAGE [45], VERIFY 3D [46, 47], ERRAT [48] and QMEAN webservers [49, 50]. Each EBV protein structure was subsequently submitted to the DaliLite webserver, with default settings. DaliLite or distance alignment matrix method server systematically scans new structures against the entire PDB for structurally similar proteins. Basically, the 3D structural coordinates of the proteins are compared by alignment of α carbon distance matrices which allows for differences in domain order, and subsequently producing a structural similarity score [39, 40]. For this study, all human proteins in the DaliLite database with z-score ≥ 2 are taken as structurally similar to the corresponding EBV protein, and are referred as hEBV proteins (human proteins structurally similar to EBV proteins).

### Prediction of EBV-host protein interactions

To identify potential human/host proteins that interact with EBV proteins, the cellular protein partners of hEBV proteins were extracted from IntAct and HPRD databases. These databases are open resources containing literature-curated molecular interactions established through in vitro and/or in vivo methods [41, 42]. The assumption here is that cellular proteins that have known interactions with hEBV proteins are possible interactants of EBV proteins owing to their structural similarity. From the resultant candidate list, only the interactions which were identified as human ribosomal proteins were selected for further investigations.

### Assessment of the predicted interactions

Predicted interactions were evaluated by comparative studies between the computational resultant dataset and a benchmark experimental dataset, in order to determine the potential reliability of the results. However, large-scale experimental data are not available for EBV-ribosomal protein interactions. To circumvent this limitation, a benchmark dataset was created by retrieving all experimental interactions between EBV and human proteins from the IntAct database, and comparing them to the EBV - human protein-protein interactions predicted in the current study.

### GO term enrichment analysis

Gene Ontology (GO) term enrichment analysis [51] of the predicted proteins was performed using the DAVID (Database for Annotation, Visualization and Integrated Discovery) tool [52, 53]. Gene Ontology (GO) is a classification scheme for consistently representing, describing and annotating gene and gene product properties, using a system of defined terms [51]. To ascertain the functional relevance of the predicted interacting proteins in this study, GO term enrichment analysis was performed using the DAVID Functional Annotation Chart tool [52, 53]. The GO chart is organized as a tree structure, wherein terms become more specific as distance from the root increases. Therefore, to achieve a good balance between specificity and coverage, GO level 3 terms were used. The *p*-values were computed with Bonferroni correction for multiple hypothesis testing and –log_10_ transformed for graphical representation of data.

### Pathway data enrichment analysis

Pathway enrichment analysis was carried out using the KEGG Orthology Based Annotation System (KOBAS 3.0) [54, 55] and DAVID. Pathway data are a primary functional source for identifying the related functions of a list of proteins [56, 57]. Significantly enriched pathways were identified using the functional set enrichment module, and adjusted by hypergeometric test with Benjamini-Hochberg False Discovery Rate (FDR) correction.

### Cellular compartment co-localization analysis

Direct interaction among two proteins requires that they share the same cellular compartment. Hence, our interaction dataset was further analysed for protein subcellular co-localization. The co-localization information for the predicted interacting proteins was assimilated based on their shared GO terms in the Cellular Compartment (CC) category. GO annotations for individual EBV proteins and human ribosomal proteins were obtained via the QuickGO tool [58]. This web-based tool allows browsing of all GO term information and GO annotations released by the Gene Ontology Annotation (GOA) project, from the UniProt Knowledgebase. Consequently, interacting pairs of EBV and human ribosomal proteins which shared at least one GO CC term were retained. The interaction network diagrams were created using Cytoscape [59].

### Molecular docking assay

Molecular docking assay for specific cases was performed to assess the reliability of our predictions, wherein the molecular and mechanistic details of interactions between EBV protein (EBNA1) and ribosomal proteins (eS10, eS25, uL10, and uL11) were evaluated. EBNA1 was chosen because it is the only viral protein expressed in all EBV-associated tumours [60]. Prior to docking analysis, 3D-models of the four RPs were generated since experimental X-ray diffraction structures are not available for the target ribosomal proteins. A three-pronged approach was used to predict their tertiary structure models. Protein sequences used for generation of 3D models were acquired from NCBI (accession no. NP_001191020.1 for eS10, NP_001019.1 for eS25, NP_444505.1 for uL10, and NP_000967.1 for uL11). For the first approach, homology modelling (or comparative modelling) to generate the 3D models was done using the SWISS-MODEL workspace [61–63]. The second approach was via a remote homology modelling technique operated by RaptorX, which uses a non-linear scoring function to combine homologous information with structural information to build 3D models [64, 65]. Lastly, a fold recognition technique (or threading technique) implemented by I-TASSER server [37, 38] was used. Selected models were geometrically refined by ModRefiner [44], and evaluated by RAMPAGE [45], VERIFY 3D [46, 47], ERRAT [48] and QMEAN webservers [49, 50].

Docking analysis of EBNA1 against each of the four ribosomal proteins was performed using two well-recognized docking tools, namely ClusPro [66, 67], and PatchDock [68]. Rigid body docking of EBNA1 - ribosomal protein in ClusPro server resulted in a file containing four categories (balanced, electrostatic-favored, hydrophobic-favored, and Van der Waals combined with electrostatics) based on the weighting of the interactions calculated. Models in all the categories were ranked by cluster size, and the best model from a highly-populated cluster in the balanced category was used. For analysis using PatchDock (with default set-up), the input parameters were the PDB format files of the proteins studied. The scoring function which considered both geometric fit and atomic desolvation energy were used to evaluate each candidate transformation. The output PDB files which presented the top 20 scoring solutions were subjected to high-throughput refinement and scoring by the FireDock server [69, 70].

The optimal EBNA1 - ribosomal protein complex from each docking assay was evaluated for best score values, and subsequently examined visually via SWISS-PdbViewer v4.1.0 [71]. For each EBNA1 - ribosomal protein complex, the root mean square deviation (RMSD) was also computed to predict the potential interaction sites based on interface contact residues (< 3.5 Å). In addition, protein-protein interactions in the binary complexes were also examined by Protein Interaction Calculator (PIC) [72].

## Additional files

Additional file 1: **Table S1.** Probable interface residues of EBNA1 and eS10 explored through the dual docking protocols. The interacting residues of EBNA1 and eS10 binding sites are indicated. **Table S2.** Predicted interfacial residues involved in hydrophobic and ionic interactions within the EBNA1-eS10 complex. (DOCX 18 kb)

Additional file 2: **Table S3.** Probable interface residues of EBNA1 and eS25 explored through the dual docking protocols. The interacting residues of EBNA1 and eS25 binding sites are indicated. **Table S4.** Predicted interfacial residues involved in hydrophobic and ionic interactions within the EBNA1-eS25 complex. (DOCX 18 kb)

Additional file 3: **Table S5.** Predicted interfacial residues of EBNA1 and uL10 explored through the dual docking protocols. The interacting residues of EBNA1 and uL10 binding sites are indicated. **Table S6.** Predicted interfacial residues involved in hydrophobic and ionic interactions within the EBNA1-uL10 complex (DOCX 18 kb)

Additional file 4: **Table S7.** Probable interfacial residues of EBNA1 and uL11 explored through the dual docking protocols. The interacting residues of EBNA1 and uL11 binding sites are indicated. **Table S8.** Predicted interfacial residues involved in hydrophobic and ionic interactions within the EBNA1-uL11 complex. (DOCX 17 kb)

## Data Availability

All data generated or analysed during this study are included in this published article (and its Additional files).

## Abbreviations

3D: Three dimensional
APC: Anaphase promoting complex
ATP2C1: Calcium-transporting ATPase type 2C member 1
BP: Biological process
CC: Cellular compartment
CD44: Homing cell adhesion molecule
CDC-20: Cell-division cycle protein 20
CSNK2A1: Casein kinase II subunit alpha
CSNK2B: Casein kinase II subunit beta
DAVID: Database for Annotation, Visualization and Integrated Discovery
EBNA1: Epstein–Barr nuclear antigen 1
EBV: Epstein-Barr Virus
EGFR: Epidermal growth factor receptor
EJC: Exon Junction Complex
EMD: Emerin
FDR: False discovery rate
GO: Gene Ontology
GOA: Gene ontology annotation
hEBV: Human proteins structurally similar to EBV proteins
HPRD: Human Protein Reference Database
KEGG: Kyoto Encyclopedia of Genes and Genomes
KOBAS: KEGG Orthology Based Annotation System
LMP: Epstein–Barr virus latent membrane protein
MDM2: Mouse double minute 2 homolog
MF: Molecular function
NCBI: National Center for Biotechnology Information
NCL: Nucleolin
NMD: Nonsense Mediated Decay
PAK: p21 activated kinase
PDB: Protein Data Bank
PDGFRB: Platelet-derived growth factor receptor beta
PIC: Protein Interaction Calculator
PPI: Protein-protein interaction
PSI-BLAST: Position-Specific Iterative BLAST
PSMA3: Proteasome subunit alpha type-3
PSME: Proteasome activator complex subunit
RABAC1: Prenylated Rab acceptor 1
RMSD: Root mean square deviation
RP: Ribosomal protein
SRPK2: Serine/threonine-protein kinase 2
TRADD: Tumor necrosis factor receptor type 1-associated death domain
UBE2I: Ubiquitin conjugating enzyme E2I
UBQLN1: Ubiquilin-1
UTR: Untranslated region
