# Supplementary material for: In silico evidence of de novo interactions between ribosomal and Epstein - Barr virus proteins
Source: BMC Mol Cell Biol. 2019 Aug 15;20:34. doi: 10.1186/s12860-019-0219-y (PMC6694676; doi:10.1186/s12860-019-0219-y)
Supplement: Supplementary file 1 — Table S1. Probable interface residues of EBNA1 and eS10 explored through the dual docking protocols. The interacting residues of EBNA1 and eS10 binding sites are indicated. Table S2. Predicted interfacial residues involved in hydrophobic and ionic interactions within the EBNA1-eS10 complex. (DOCX 18 kb) [file 12860_2019_219_MOESM1_ESM.docx]

Additional file 1

**Table S1** Probable interface residues of EBNA1 and eS10 explored through the dual docking protocols. The interacting residues of EBNA1 and eS10 binding sites are indicated.

| **Protein** | **Docking servers** | **Interface residues** |
| --- | --- | --- |
| EBNA1 | ClusPro | Arg532, Ala533, Tyr561, Met563, Val564, Phe565, Leu566, Thr568, Ala588, Pro589, Cys591, Ile593, Arg594, Asp602, Val604, Asp605, Leu606, Trp609 |
|  | PatchDock/FireDock | Arg43, Thr85, His86, Gly122, Gly123, Gly155, Gly156, Ala157, Gly158, Ala159, Gly160, Gly161, Gly174, Ala196, Gly197, Ala198, Gly199, Gly200, Gly201, Ala218, Ala220, Gly239, Ala240, Gly241, Ala242, Gly243, Gly244, Ala245, Gly246, Ala247, Gly248 |
| eS10 | ClusPro | Glu18, Gln61, Ala63, Trp64, His66, Trp68, Arg95, Arg96, Ser97, Glu100, Pro114, Arg116, Arg119, Gly159, Arg160, Gly161, Gln162, Pro163, Pro164, Glu165 |
|  | PatchDock/FireDock | Tyr12, Met46, Lys47, Ala48, Met49, Gly50, Ser51, Glu52, Lys53, Ser54, Arg55, Gly56, Tyr57, Val58, Lys59, Glu60, Gln61, Phe62, Phe67, Tyr68, Trp69, Leu71, Thr72, Asn73, Glu74, Gln77, Tyr78, Leu117, Arg119, Gly120, Glu121 |

**Table S2** Predicted interfacial residues involved in hydrophobic and ionic interactions within the EBNA1-eS10 complex.

| **Hydrophobic Interactions** | | | | | | | | | |
| --- | --- | --- | --- | --- | --- | --- | --- | --- | --- |
| **Position** | **Residue** | **Chain** | | **Position** | | **Residue** | | **Chain** | |
| 124 | Ala | EBNA1 | | 127 | | Tyr | | eS10 | |
| 157 | Ala | EBNA1 | | 58 | | Val | | eS10 | |
| 159 | Ala | EBNA1 | | 117 | | Leu | | eS10 | |
| 198 | Ala | EBNA1 | | 58 | | Val | | eS10 | |
| 198 | Ala | EBNA1 | | 69 | | Trp | | eS10 | |
| 218 | Ala | EBNA1 | | 62 | | Phe | | eS10 | |
| 220 | Ala | EBNA1 | | 63 | | Ala | | eS10 | |
| 245 | Ala | EBNA1 | | 52 | | Leu | | eS10 | |
| **Ionic Interactions** | | | | | | | | | |
| **Position** | **Residue** | | **Chain** | | **Position** | | **Residue** | | **Chain** |
| 86 | His | | EBNA1 | | 81 | | Asp | | eS10 |
