# Supplementary material for: In silico evidence of de novo interactions between ribosomal and Epstein - Barr virus proteins
Source: BMC Mol Cell Biol. 2019 Aug 15;20:34. doi: 10.1186/s12860-019-0219-y (PMC6694676; doi:10.1186/s12860-019-0219-y)
Supplement: Supplementary file 2 — Table S3. Probable interface residues of EBNA1 and eS25 explored through the dual docking protocols. The interacting residues of EBNA1 and eS25 binding sites are indicated. Table S4. Predicted interfacial residues involved in hydrophobic and ionic interactions within the EBNA1-eS25 complex. (DOCX 18 kb) [file 12860_2019_219_MOESM2_ESM.docx]

**Additional file 2**

**Table S3** Probable interface residues of EBNA1 and eS25 explored through the dual docking protocols. The interacting residues of EBNA1 and eS25 binding sites are indicated.

| **Protein** | **Docking servers** | **Interface residues** |
| --- | --- | --- |
| EBNA1 | ClusPro | Tyr561, Met563, Phe565, Leu566, Gln567, Arg594, Val604, Asp605, Trp609 |
|  | PatchDock/FireDock | Gly452, Gln453, Gly473, Leu488, Leu489 |
| eS25 | ClusPro | Met1, Pro3, Lys4, Asp5, Val48, Leu49, Phe50, Asp51, Ser82, Leu83 |
|  | PatchDock/FireDock | Ser36, Lys37, Gly38, Lys39, Val40, Arg41, Leu44 |

**Table S4** Predicted interfacial residues involved in hydrophobic and ionic interactions within the EBNA1-eS25 complex.

| **Hydrophobic Interactions** | | | | | |
| --- | --- | --- | --- | --- | --- |
| **Position** | **Residue** | **Chain** | **Position** | **Residue** | **Chain** |
| 533 | Leu | EBNA1 | 1 | Met | eS25 |
| 549 | Pro | EBNA1 | 1 | Met | eS25 |
| 561 | Tyr | EBNA1 | 1 | Met | eS25 |
| 563 | Met | EBNA1 | 47 | Leu | eS25 |
| 563 | Met | EBNA1 | 48 | Val | eS25 |
| 564 | Val | EBNA1 | 49 | Leu | eS25 |
| 565 | Phe | EBNA1 | 1 | Met | eS25 |
| 565 | Phe | EBNA1 | 2 | Pro | eS25 |
| 565 | Phe | EBNA1 | 47 | Leu | eS25 |
| 566 | Leu | EBNA1 | 2 | Pro | eS25 |
| 566 | Leu | EBNA1 | 53 | Ala | eS25 |
| 593 | Ile | EBNA1 | 55 | Tyr | eS25 |
| 600 | Phe | EBNA1 | 49 | Leu | eS25 |
| 604 | Val | EBNA1 | 48 | Val | eS25 |
| 606 | Leu | EBNA1 | 48 | Val | eS25 |
| 606 | Leu | EBNA1 | 49 | Leu | eS25 |
| 606 | Leu | EBNA1 | 83 | Leu | eS25 |
| 609 | Trp | EBNA1 | 49 | Leu | eS25 |
| 609 | Trp | EBNA1 | 50 | Phe | eS25 |
| 609 | Trp | EBNA1 | 83 | Leu | eS25 |
