# Supplementary material for: In silico evidence of de novo interactions between ribosomal and Epstein - Barr virus proteins
Source: BMC Mol Cell Biol. 2019 Aug 15;20:34. doi: 10.1186/s12860-019-0219-y (PMC6694676; doi:10.1186/s12860-019-0219-y)
Supplement: Supplementary file 3 — Table S5. Predicted interfacial residues of EBNA1 and uL10 explored through the dual docking protocols. The interacting residues of EBNA1 and uL10 binding sites are indicated. Table S6. Predicted interfacial residues involved in hydrophobic and ionic interactions within the EBNA1-uL10 complex (DOCX 18 kb) [file 12860_2019_219_MOESM3_ESM.docx]

**Additional file 3**

**Table S5** Predicted interfacial residues of EBNA1 and uL10 explored through the dual docking protocols. The interacting residues of EBNA1 and uL10 binding sites are indicated.

| **Protein** | **Docking servers** | **Interface residues** |
| --- | --- | --- |
| EBNA1 | ClusPro | Gly15, Arg33, Arg34, Lys75, Arg76, Pro77, Ala95, Gly256, Ala280, Gly296, Gly298, Gly299, Ala322, Gly328, Arg329, Arg339, Arg353, Arg354, Arg370, Arg372, Arg374, Arg376, Gln387, Glu573, Phe571, Lys586, Glu615, Gly621, Asp622 |
|  | PatchDock/FireDock | Arg532, Leu533 |
| uL10 | ClusPro | Ala113, Ser170, Thr173, Thr285, Thr286, Ala287, Ala288, Pro289, Ala290, Ala291, Ala292, Ala293, Ala294, Pro295, Ala296, Lis297, Val298, Ala300, Glu302, Glu303, Glu305, Glu309, Asp310, Met311, Gly312, Phe313, Gly314, Leu315, Phe316, Asp317 |
|  | PatchDock/FireDock | Arg6, Lys16 |

**Table S6** Predicted interfacial residues involved in hydrophobic and ionic interactions within the EBNA1-uL10 complex

| **Hydrophobic Interactions** | | | | | | | | | |
| --- | --- | --- | --- | --- | --- | --- | --- | --- | --- |
| **Position** | **Residue** | **Chain** | | **Position** | | **Residue** | | **Chain** | |
| 77 | Pro | EBNA1 | | 316 | | Phe | | uL10 | |
| 118 | Ala | EBNA1 | | 315 | | Leu | | uL10 | |
| 255 | Ala | EBNA1 | | 313 | | Phe | | uL10 | |
| 280 | Ala | EBNA1 | | 313 | | Phe | | uL10 | |
| 297 | Ala | EBNA1 | | 311 | | Met | | uL10 | |
| 539 | Leu | EBNA1 | | 291 | | Ala | | uL10 | |
| 539 | Leu | EBNA1 | | 292 | | Ala | | uL10 | |
| 570 | Ile | EBNA1 | | 296 | | Ala | | uL10 | |
| 570 | Ile | EBNA1 | | 298 | | Val | | uL10 | |
| 571 | Phe | EBNA1 | | 292 | | Ala | | uL10 | |
| 571 | Phe | EBNA1 | | 293 | | Ala | | uL10 | |
| 571 | Phe | EBNA1 | | 294 | | Ala | | uL10 | |
| 574 | Val | EBNA1 | | 291 | | Ala | | uL10 | |
| 587 | Pro | EBNA1 | | 298 | | Val | | uL10 | |
| 618 | Ala | EBNA1 | | 293 | | Ala | | uL10 | |
| 619 | Ala | EBNA1 | | 290 | | Ala | | uL10 | |
| **Ionic Interactions** | | | | | | | | | |
| **Position** | **Residue** | | **Chain** | | **Position** | | **Residue** | | **Chain** |
| 33 | Arg | | EBNA1 | | 299 | | Glu | | uL10 |
| 33 | Arg | | EBNA1 | | 302 | | Glu | | uL10 |
| 33 | Arg | | EBNA1 | | 309 | | Glu | | uL10 |
| 34 | Arg | | EBNA1 | | 310 | | Asp | | uL10 |
| 75 | Lys | | EBNA1 | | 302 | | Glu | | uL10 |
| 76 | Arg | | EBNA1 | | 303 | | Glu | | uL10 |
| 76 | Arg | | EBNA1 | | 305 | | Glu | | uL10 |
| 76 | Arg | | EBNA1 | | 317 | | Asp | | uL10 |
| 370 | Arg | | EBNA1 | | 309 | | Glu | | uL10 |
