# Supplementary material for: In silico evidence of de novo interactions between ribosomal and Epstein - Barr virus proteins
Source: BMC Mol Cell Biol. 2019 Aug 15;20:34. doi: 10.1186/s12860-019-0219-y (PMC6694676; doi:10.1186/s12860-019-0219-y)
Supplement: Supplementary file 4 — Table S7. Probable interfacial residues of EBNA1 and uL11 explored through the dual docking protocols. The interacting residues of EBNA1 and uL11 binding sites are indicated. Table S8. Predicted interfacial residues involved in hydrophobic and ionic interactions within the EBNA1-uL11 complex. (DOCX 17 kb) [file 12860_2019_219_MOESM4_ESM.docx]

**Additional file 4**

**Table S7** Probable interfacial residues of EBNA1 and uL11 explored through the dual docking protocols. The interacting residues of EBNA1 and uL11 binding sites are indicated.

| **Protein** | **Docking servers** | **Interfacial residues in** |
| --- | --- | --- |
| EBNA1 | ClusPro | Asp499, Glu500, Phe541, Gly542, Ala544, Leu554, Glu556, Val559, Cys560, Asp577, Ala578, Ile579, Asp601, Asp602, Asp605 |
|  | PatchDock/FireDock | Gly239, Gly241, Ala242, Gly243, Gly246, Ala247, Gly248, Gly249, Ala289, Gly309 |
| uL11 | ClusPro | Tyr14, Lys31, Ser76, Arg114, Gln115, Arg117, His118, Arg119, Leu121, Ala122, Arg123 |
|  | PatchDock/FireDock | Asp153, Asp154, Ser157, Gly158, Ala159, Val160, Glu161, Cys162 |

**Table S8** Predicted interfacial residues involved in hydrophobic and ionic interactions within the EBNA1-uL11 complex

| **Hydrophobic Interactions** | | | | | | | | | |
| --- | --- | --- | --- | --- | --- | --- | --- | --- | --- |
| **Position** | **Residue** | **Chain** | | **Position** | | **Residue** | | **Chain** | |
| 558 | Ile | EBNA1 | | 14 | | Tyr | | uL11 | |
| 561 | Tyr | EBNA1 | | 12 | | Val | | uL11 | |
| 561 | Tyr | EBNA1 | | 14 | | Tyr | | uL11 | |
| 562 | Phe | EBNA1 | | 121 | | Leu | | uL11 | |
| 604 | Val | EBNA1 | | 13 | | Val | | uL11 | |
| **Ionic Interactions** | | | | | | | | | |
| **Position** | **Residue** | | **Chain** | | **Position** | | **Residue** | | **Chain** |
| 499 | Asp | | EBNA1 | | 119 | | Arg | | uL11 |
| 500 | Glu | | EBNA1 | | 119 | | Arg | | uL11 |
| 577 | Asp | | EBNA1 | | 114 | | Arg | | uL11 |
| 581 | Asp | | EBNA1 | | 118 | | His | | uL11 |
| 601 | Asp | | EBNA1 | | 117 | | Arg | | uL11 |
| 602 | Asp | | EBNA1 | | 117 | | Arg | | uL11 |
| 602 | Asp | | EBNA1 | | 31 | | Lys | | uL11 |
| 605 | Asp | | EBNA1 | | 31 | | Lys | | uL11 |
